# Generalizations on Some Hermite-Hadamard Type Inequalities for Differentiable Convex Functions with Applications to Weighted Means

**DOI:** 10.1155/2014/717164

**Published:** 2014-01-16

**Authors:** Banyat Sroysang

**Affiliations:** Department of Mathematics and Statistics, Faculty of Science and Technology, Thammasat University, Pathum Thani 12121, Thailand

## Abstract

Some new Hermite-Hadamard type inequalities for differentiable convex functions were presented by Xi and Qi. In this paper, we present new generalizations on the Xi-Qi inequalities.

## 1. Introduction

The Hermite-Hadamard inequality [1–3] states that if *f* is a convex function on [*a*, *b*], then

(1) $$\begin{array}{c} f\left(\frac{a+b}{2}\right)\leq \frac{1}{b-a}\int_{a}^{b}f\left(x\right)dx\leq \frac{f\left(a\right)+f\left(b\right)}{2}. \end{array}$$

Let *f* : *I*⊆ℝ → ℝ be differentiable on *I*° and {*a*, *b*}⊆*I* with *a* < *b*. Below we recall some Hermite-Hadamard type inequalities.

In 1998, Dragomir and Agarwal [4] showed that (i) if |*f*′(*x*)| is convex on [*a*, *b*], then

(2) |f(a)+f(b)2−1b−a∫abf(x)dx|  ≤(b−a)(|f′(a)|+|f′(b)|)8,

and (ii) if |*f*′(*x*)|^*p*/(*p*−1)^ is convex on [*a*, *b*] with *p* > 1, then

(3) |f(a)+f(b)2−1b−a∫abf(x)dx| ≤b−a2(p+1)1/p  ×(|f′(a)|p/(p−1)+|f′(b)|p/(p−1)2)(p−1)/p.

In 2000, Pearce and Pečarić [5] showed that if |*f*′(*x*)|^*q*^ is convex on [*a*, *b*] with *q* ≥ 1, then

(4) |f(a)+f(b)2−1b−a∫abf(x)dx|  ≤b−a4(|f′(a)|q+|f′(b)|q2)1/q,|f(a+b2)−1b−a∫abf(x)dx|  ≤b−a4(|f′(a)|q+|f′(b)|q2)1/q.

In 2004, Kirmaci [6] showed that if |*f*′(*x*)|^*p*/(*p*−1)^ is convex on [*a*, *b*] with *p* > 1, then

(5) |f(a+b2)−1b−a∫abf(x)dx|  ≤b−a4(4p+1)1/p(|f′(a)|+|f′(b)|),|f(a+b2)−1b−a∫abf(x)dx|≤b−a16(4p+1)1/p ×(|f′(a)|p/(p−1)+3|f′(b)|p/(p−1))(p−1)/p +b−a16(4p+1)1/p ×(3|f′(a)|p/(p−1)+|f′(b)|p/(p−1))(p−1)/p.

In 2010, Sarikaya et al. [7] showed that if *f*′ ∈ *L*[*a*, *b*] and |*f*′(*x*)|^*q*^ is convex on [*a*, *b*] with *q* ≥ 1, then

(6) |16[f(a)+f(b)+4f(a+b2)]−1b−a∫abf(x)dx|≤b−a12(2q+1+13(q+1))1/q ×[(3|f′(a)|q+|f′(b)|q4)1/q   + (|f′(a)|q+3|f′(b)|q4)1/q],|16[f(a)+f(b)+4f(a+b2)]−1b−a∫abf(x)dx|≤5(b−a)72 ×[(61|f′(a)|q+29|f′(b)|q90)1/q   + (29|f′(a)|q+61|f′(b)|q90)1/q].

In 2012, Xi and Qi [8] showed that if *λ*, *μ* ∈ [0,1] and if *f*′ ∈ *L*[*a*, *b*] and |*f*′(*x*)|^*q*^ is convex on [*a*, *b*] with *q* ≥ 1, then

(7) |λf(a)+μf(b)2+2−λ−μ2f(a+b2)−1b−a∫abf(x)dx|≤b−a8(1−2λ+2λ2)1−1/q ×[((4−9λ+12λ2−2λ3)|f′(a)|q    +(2−3λ+2λ3)|f′(b)|q)×(6)−1]1/q +b−a8(1−2μ+2μ2)1−1/q ×[((2−3μ+2μ3)|f′(a)|q    +(4−9μ+12μ2−2μ3)|f′(b)|q)×(6)−1]1/q,|λf(a)+μf(b)2+2−λ−μ2f(a+b2)−1b−a∫abf(x)dx|≤b−a4(12(q+1)(q+2))1/q ×[((q+3−λ)(1−λ)q+1+(2q+4−λ)λq+1)|f′(a)|q   +((q+1+λ)(1−λ)q+1+λq+2)|f′(b)|q]1/q +b−a4(12(q+1)(q+2))1/q ×[((q+1+μ)(1−μ)q+1+μq+2)|f′(a)|q   +((q+3−μ)(1−μ)q+1+(2q+4−μ)μq+1)   ×|f′(b)|q]1/q.

Moreover, for other results involving the Hermite-Hadamard type inequalities, we also refer to [9–23].

In this paper, we generalize the Xi-Qi inequalities.

## 2. Preliminaries

**Lemma 1**

Let *λ*,  *μ* ∈ ℝ and let *f* : *I*⊆ℝ → ℝ be differentiable on *I*° and {*a*, *b*}⊆*I* with *a* < *b*. Assume that *f*′ ∈ *L*[*a*, *b*] and 0 < *ϵ* < *b* − *a*. Then

(8) ϵλf(a)+(b−a−ϵ)μf(b) +[ϵ(1−λ)+(b−a−ϵ)(1−μ)]f(a+ϵ)−∫abf(x)dx  =∫01[ϵ2(1−λ−t)f′(ta+(1−t)(a+ϵ))      + (b−a−ϵ)2(μ−t)]     ×f′(t(a+ϵ)+(1−t)b)dt.

**Proof**

Integrating by part and changing variable, we have

(9) ∫01ϵ2(1−λ−t)f′(ta+(1−t)(a+ϵ))dt =∫t=0t=1ϵ(λ+t−1)df(ta+(1−t)(a+ϵ)) =[ϵ(λ+t−1)f(ta+(1−t)(a+ϵ))]t=0t=1  −ϵ∫01f(ta+(1−t)(a+ϵ))dt =ϵλf(a)+ϵ(1−λ)f(a+ϵ)−∫aa+ϵf(x)dx,∫01(b−a−ϵ)2(μ−t)f′(t(a+ϵ)+(1−t)b)dt =∫t=0t=1(b−a−ϵ)(t−μ)df(t(a+ϵ)+(1−t)b) =[(b−a−ϵ)(t−μ)f(t(a+ϵ)+(1−t)b)]t=0t=1  −(b−a−ϵ)∫01f(t(a+ϵ)+(1−t)b)dt =(b−a−ϵ)(1−μ)f(a+ϵ)  +(b−a−ϵ)μf(b)−∫a+ϵbf(x)dx.

Thus,

(10) ∫01[ϵ2(1−λ−t)f′(ta+(1−t)(a+ϵ))  + (b−a−ϵ)2(μ−t)]f′(t(a+ϵ)+(1−t)b)dt=ϵλf(a)+(b−a−ϵ)μf(b) +[ϵ(1−λ)+(b−a−ϵ)(1−μ)] ×f(a+ϵ)−∫abf(x)dx.

**Lemma (see [8])**

Let *s* > 0 and 0 ≤ *ξ* ≤ 1. Then

(11) $$\begin{array}{c} \int_{0}^{1}{\left|\xi -t\right|}^{s}dt=\frac{\xi^{s+1}+{\left(1-\xi \right)}^{s+1}}{s+1},\\ \int_{0}^{1}t{\left|\xi -t\right|}^{s}dt=\frac{\xi^{s+2}+\left(s+1+\xi \right){\left(1-\xi \right)}^{s+1}}{\left(s+1\right)\left(s+2\right)}. \end{array}$$

## 3. Main Results

**Theorem 3**

Let *λ*,  *μ* ∈ [0,1] and let *f* : *I*⊆ℝ → ℝ be differentiable on *I*° and {*a*, *b*}⊆*I* with *a* < *b*. Assume that *f*′ ∈ *L*[*a*, *b*] and 0 < *ϵ* < *b* − *a*. If |*f*′(*x*)|^*q*^ is convex on [*a*, *b*] with *q* ≥ 1, then

(12) |ϵλf(a)+(b−a−ϵ)μf(b) +[ϵ(1−λ)+(b−a−ϵ)(1−μ)] ×f(a+ϵ)−∫abf(x)dx|≤ϵ2((1−λ)2+λ22)1−(1/q) ×{16[3−6λ+6λ2−ϵb−a(2−3λ+2λ3)]|f′(a)|q    +16[ϵb−a(2−3λ+2λ3)]|f′(b)|q}1/q +(b−a−ϵ)2(μ2+(1−μ)22)1−(1/q) ×{16[(1−ϵb−a)(2−3μ+2μ3)]|f′(a)|q    +16[1−3μ+6μ2−2μ3+ϵb−a(2−3μ+2μ3)]    ×|f′(b)|q}1/q.

**Proof**

Suppose that |*f*′(*x*)|^*q*^ is convex on [*a*, *b*] with *q* ≥ 1. By Lemma 1, we have

(13) |ϵλf(a)+(b−a−ϵ)μf(b) +[ϵ(1−λ)+(b−a−ϵ)(1−μ)] ×f(a+ϵ)−∫abf(x)dx|≤ϵ2∫01|1−λ−t|          ×|f′(ta+(1−t)(a+ϵ))|dt +(b−a−ϵ)2∫01|μ−t|          ×|f′(t(a+ϵ)+(1−t)b)|dt.

*Case*  (*q* = 1). By the convexity of |*f*′(*x*)| and Lemma 2, we have

(14) ∫01|1−λ−t||f′(ta+(1−t)(a+ϵ))|dt=∫01|1−λ−t|   ×|f′((1−ϵ(1−t)b−a)a+ϵ(1−t)b−ab)|dt≤∫01|1−λ−t|   ×[(1−ϵ(1−t)b−a)|f′(a)|+ϵ(1−t)b−a|f′(b)|]dt=[(1−ϵb−a)|f′(a)|+ϵb−a|f′(b)|] ×∫01|1−λ−t|dt   +[ϵb−a|f′(a)|−ϵb−a|f′(b)|] ×∫01t|1−λ−t|dt   =[(1−ϵb−a)|f′(a)|+ϵb−a|f′(b)|] ×(1−λ)2+λ22 +[ϵb−a|f′(a)|−ϵb−a|f′(b)|] ×(1−λ)3+(3−λ)λ26=16[3−6λ+6λ2−ϵb−a(2−3λ+2λ3)]|f′(a)| +16[ϵb−a(2−3λ+2λ3)]|f′(b)|,∫01|μ−t||f′(t(a+ϵ)+(1−t)b)|dt=∫01|μ−t|  ×|f′((t−ϵtb−a)a+(1−(t−ϵtb−a))b)|dt≤∫01|μ−t|[(t−ϵtb−a)|f′(a)|        +(1−(t−ϵtb−a))|f′(b)|]dt=[(1−ϵb−a)|f′(a)|−(1−ϵb−a)|f′(b)|] ×∫01t|μ−t|dt   +|f′(b)|∫01|μ−t|dt=[(1−ϵb−a)|f′(a)|−(1−ϵb−a)|f′(b)|] ×μ3+(2+μ)(1−μ)26 +|f′(b)|μ2+(1−μ)22=16[(1−ϵb−a)(2−3μ+2μ3)]|f′(a)| +16[1−3μ+6μ2−2μ3+ϵb−a(2−3μ+2μ3)] ×|f′(b)|.

Thus,

(15) |ϵλf(a)+(b−a−ϵ)μf(b) +[ϵ(1−λ)+(b−a−ϵ)(1−μ)] × f(a+ϵ)−∫abf(x)dx|≤ϵ26[3−6λ+6λ2−ϵb−a(2−3λ+2λ3)]|f′(a)| +ϵ26[ϵb−a(2−3λ+2λ3)]|f′(b)| +(b−a−ϵ)26 ×[(1−ϵb−a)(2−3μ+2μ3)]|f′(a)| +(b−a−ϵ)26 ×[1−3μ+6μ2−2μ3+ϵb−a(2−3μ+2μ3)]|f′(b)|.

*Case*  (*q* > 1). By Hölder's inequality, we have

(16) |ϵλf(a)+(b−a−ϵ)μf(b) +[ϵ(1−λ)+(b−a−ϵ)(1−μ)] × f(a+ϵ)−∫abf(x)dx|≤ϵ2(∫01|1−λ−t|dt)1−(1/q) ×(∫01|1−λ−t||f′(ta+(1−t)(a+ϵ))|qdt)1/q +(b−a−ϵ)2(∫01|μ−t|dt)1−(1/q) ×(∫01|μ−t||f′(t(a+ϵ)+(1−t)b)|qdt)1/q=ϵ2((1−λ)2+λ22)1−(1/q) ×(∫01|1−λ−t||f′(ta+(1−t)(a+ϵ))|qdt)1/q +(b−a−ϵ)2(μ2+(1−μ)22)1−(1/q) ×(∫01|μ−t||f′(t(a+ϵ)+(1−t)b)|qdt)1/q.

By the convexity of |*f*′(*x*)|^*q*^ and Lemma 2, we have

(17) ∫01|1−λ−t||f′(ta+(1−t)(a+ϵ))|qdt=∫01|1−λ−t||f′((1−ϵ(1−t)b−a)a+ϵ(1−t)b−ab)|qdt≤∫01|1−λ−t|  ×[(1−ϵ(1−t)b−a)|f′(a)|q+ϵ(1−t)b−a|f′(b)|q]dt=[(1−ϵb−a)|f′(a)|q+ϵb−a|f′(b)|q]∫01|1−λ−t|dt +[ϵb−a|f′(a)|q−ϵb−a|f′(b)|q]∫01t|1−λ−t|dt=[(1−ϵb−a)|f′(a)|q+ϵb−a|f′(b)|q](1−λ)2+λ22 +[ϵb−a|f′(a)|q−ϵb−a|f′(b)|q](1−λ)3+(3−λ)λ26=16[3−6λ+6λ2−ϵb−a(2−3λ+2λ3)]|f′(a)|q +16[ϵb−a(2−3λ+2λ3)]|f′(b)|q,∫01|μ−t||f′(t(a+ϵ)+(1−t)b)|qdt=∫01|μ−t||f′((t−ϵtb−a)a+(1−(t−ϵtb−a))b)|qdt≤∫01|μ−t|   ×[(t−ϵtb−a)|f′(a)|q     +(1−(t−ϵtb−a))|f′(b)|q]dt=[(1−ϵb−a)|f′(a)|q−(1−ϵb−a)|f′(b)|q] ×∫01t|μ−t|dt   +|f′(b)|q∫01|μ−t|dt=[(1−ϵb−a)|f′(a)|q−(1−ϵb−a)|f′(b)|q] ×μ3+(2+μ)(1−μ)26 +|f′(b)|q(μ2+(1−μ)22)=16[(1−ϵb−a)(2−3μ+2μ3)]|f′(a)|q +16[1−3μ+6μ2−2μ3+ϵb−a(2−3μ+2μ3)] ×|f′(b)|q.

Thus,

(18) |ϵλf(a)+(b−a−ϵ)μf(b) +[ϵ(1−λ)+(b−a−ϵ)(1−μ)] ×f(a+ϵ)−∫abf(x)dx|≤ϵ2((1−λ)2+λ22)1−(1/q) ×{16[3−6λ+6λ2−ϵb−a(2−3λ+2λ3)]    × |f′(a)|q+16[ϵb−a(2−3λ+2λ3)]|f′(b)|q}1/q +(b−a−ϵ)2(μ2+(1−μ)22)1−(1/q) ×{16[(1−ϵb−a)(2−3μ+2μ3)]|f′(a)|q   +16[1−3μ+6μ2−2μ3+ϵb−a(2−3μ+2μ3)]   ×|f′(b)|q}1/q.

This proof is completed.

It is easy to notice that if we put *ϵ* = (*b* − *a*)/2 in Theorem 3 then we get the following.

**Corollary 4 (see [8])**

Let *λ*,  *μ* ∈ [0,1] and let *f* : *I*⊆ℝ → ℝ be differentiable on *I*° and {*a*, *b*}⊆*I* with *a* < *b*. Assume that *f*′ ∈ *L*[*a*, *b*]. If |*f*′(*x*)|^*q*^ is convex on [*a*, *b*] with *q* ≥ 1, then

(19) |λf(a)+μf(b)2+2−λ−μ2f(a+b2)−1b−a∫abf(x)dx|≤b−a8(1−2λ+2λ2)1−1/q ×[((4−9λ+12λ2−2λ3)|f′(a)|q    +(2−3λ+2λ3)|f′(b)|q)×(6)−1]1/q +b−a8(1−2μ+2μ2)1−1/q ×[((2−3μ+2μ3)|f′(a)|q    +(4−9μ+12μ2−2μ3)|f′(b)|q)×(6)−1]1/q.

One can easily check that if we put *ϵ* = (*b* − *a*)/3 in Theorem 3, then we get the following.

**Corollary 5**

Let *λ*,  *μ* ∈ [0,1] and let *f* : *I*⊆ℝ → ℝ be differentiable on *I*° and {*a*, *b*}⊆*I* with *a* < *b*. Assume that *f*′ ∈ *L*[*a*, *b*]. If |*f*′(*x*)|^*q*^ is convex on [*a*, *b*] with *q* ≥ 1, then

(20) |λf(a)+2μf(b)3+(1−λ3−2μ3)f(2a+b3) − 1b−a∫abf(x)dx|≤b−a9((1−λ)2+λ22)1−(1/q) ×(118(7−15λ+18λ2−2λ3)|f′(a)|q    + 118(2−3λ+2λ3)|f′(b)|q)1/q +4(b−a)9(μ2+(1−μ)22)1−(1/q) ×(19(2−3μ+2μ3)|f′(a)|q    +118(5−12μ+18μ2−4μ3)|f′(b)|q)1/q.

One can easily check that if we put *ϵ* = 2(*b* − *a*)/3 in Theorem 3 then we get the following.

**Corollary 6**

Let *λ*,  *μ* ∈ [0,1] and let *f* : *I*⊆ℝ → ℝ be differentiable on *I*° and {*a*, *b*}⊆*I* with *a* < *b*. Assume that *f*′ ∈ *L*[*a*, *b*]. If |*f*′(*x*)|^*q*^ is convex on [*a*, *b*] with *q* ≥ 1, then

(21) |2λf(a)+μf(b)3+(1−2λ3−μ3)f(a+2b3) −1b−a∫abf(x)dx|≤4(b−a)9((1−λ)2+λ22)1−(1/q) ×(118(5−12λ+18λ2−4λ3)|f′(a)|q    +19(2−3λ+2λ3)|f′(b)|q)1/q +b−a9(μ2+(1−μ)22)1−(1/q) ×(118(2−3μ+2μ3)|f′(a)|q    + 118(7−15μ+18μ2−2μ3)|f′(b)|q)1/q.

It is easy to notice that if we put *λ* = *μ* = 0 in Theorem 3 then we get the following.

**Corollary 7**

Let *f* : *I*⊆ℝ → ℝ be differentiable on *I*° and {*a*, *b*}⊆*I* with *a* < *b*. Assume that *f*′ ∈ *L*[*a*, *b*] and 0 < *ϵ* < *b* − *a*. If |*f*′(*x*)|^*q*^ is convex on [*a*, *b*] with *q* ≥ 1, then

(22) 21−(1/q)(b−a)|f(a+ϵ)−1b−a∫abf(x)dx|≤ϵ2((12−ϵ3(b−a))|f′(a)|q+ϵ3(b−a)|f′(b)|q)1/q +(b−a−ϵ)2 ×((13−ϵ3(b−a))|f′(a)|q   +(16+ϵ3(b−a))|f′(b)|q)1/q.

It is easy to notice that if we put *λ* = *μ* = 1/2 in Theorem 3 then we get the following.

**Corollary 8**

Let *f* : *I*⊆ℝ → ℝ be differentiable on *I*° and {a, *b*}⊆*I* with *a* < *b*. Assume that *f*′ ∈ *L*  [*a*, *b*] and 0 < *ϵ* < *b* − *a*. If |*f*′(*x*)|^*q*^ is convex on [*a*, *b*] with *q* ≥ 1, then

(23) 41−(1/q)|ϵf(a)+(b−a−ϵ)f(b)+(b−a)f(a+ϵ)2    −∫abf(x)dx|≤ϵ2((14−ϵ8(b−a))|f′(a)|q+ϵ8(b−a)|f′(b)|q)1/q +(b−a−ϵ)2 ×((18−ϵ8(b−a))|f′(a)|q   +(18+ϵ8(b−a))|f′(b)|q)1/q.

It is easy to notice that if we put *λ* = *μ* = 1 in Theorem 3 then we get the following.

**Corollary 9**

Let *f* : *I*⊆ℝ → ℝ be differentiable on *I*° and {*a*, *b*}⊆*I* with *a* < *b*. Assume that *f*′ ∈ *L*[*a*, *b*] and 0 < *ϵ* < *b* − *a*. If |*f*′(*x*)|^*q*^ is convex on [*a*, *b*] with *q* ≥ 1, then

(24) 21−(1/q)|ϵf(a)+(b−a−ϵ)f(b)−∫abf(x)dx|≤ϵ2((13−ϵ6(b−a))|f′(a)|q+ϵ6(b−a)|f′(b)|q)1/q +(b−a−ϵ)2 ×((16−ϵ6(b−a))|f′(a)|q   +(13+ϵ6(b−a))|f′(b)|q)1/q.

**Theorem 10**

Let *λ*,  *μ* ∈ [0,1] and let *f* : *I*⊆ℝ → ℝ be differentiable on *I*° and {*a*, *b*}⊆*I* with *a* < *b*. Assume that *f*′ ∈ *L*[*a*, *b*] and 0 < *ϵ* < *b* − *a*. If |*f*′(*x*)|^*q*^ is convex on [*a*, *b*] with *q* ≥ 1, then

(25) |ϵλf(a)+(b−a−ϵ)μf(b) +[ϵ(1−λ)+(b−a−ϵ)(1−μ)] ×f(a+ϵ)−∫abf(x)dx|≤ϵ2{[(1−λ)q+1+λq+1q+1−(ϵb−a)     ×(1+λ+q)(1−λ)q+1+λq+2(q+1)(q+2)]|f′(a)|q     +[(ϵb−a)(1+λ+q)(1−λ)q+1+λq+2(q+1)(q+2)]    × |f′(b)|q}1/q +(b−a−ϵ)2 ×{[(1−ϵb−a)μq+2+(q+1+μ)(1−μ)q+1(q+1)(q+2)]|f′(a)|q    +[μq+1+(1−μ)q+1q+1−(1−ϵb−a)      ×μq+2+(q+1+μ)(1−μ)q+1(q+1)(q+2)]    ×|f′(b)|q}1/q.

**Proof**

Suppose that |*f*′(*x*)|^*q*^ is convex on [*a*, *b*] with *q* ≥ 1. If *q* = 1, then, by Theorem 3, we have

(26) |ϵλf(a)+(b−a−ϵ)μf(b) +[ϵ(1−λ)+(b−a−ϵ)(1−μ)] × f(a+ϵ)−∫abf(x)dx|≤ϵ2{16[3−6λ+6λ2−ϵb−a(2−3λ+2λ3)]|f′(a)|   + 16[ϵb−a(2−3λ+2λ3)]|f′(b)|} +(b−a−ϵ)2 ×{16[(1−ϵb−a)(2−3μ+2μ3)]|f′(a)|   +16[1−3μ+6μ2−2μ3+ϵb−a(2−3μ+2μ3)]   ×|f′(b)|}=ϵ2{[(1−λ)2+λ22−(ϵb−a)(2+λ)(1−λ)2+λ36]   ×|f′(a)|+[(ϵb−a)(2+λ)(1−λ)2+λ36]   ×|f′(b)|} +(b−a−ϵ)2 ×{[(1−ϵb−a)μ3+(2+μ)(1−μ)26]|f′(a)|   +[μ2+(1−μ)22−(1−ϵb−a)     ×μ3+(2+μ)(1−μ)26]|f′(b)|}.

Next, we suppose that *q* > 1. By Lemma 1 and Hölder's inequality, we have

(27) |ϵλf(a)+(b−a−ϵ)μf(b) +[ϵ(1−λ)+(b−a−ϵ)(1−μ)] ×f(a+ϵ)−∫abf(x)dx|≤ϵ2∫01|1−λ−t||f′(ta+(1−t)(a+ϵ))|dt +(b−a−ϵ)2∫01|μ−t||f′(t(a+ϵ)+(1−t)b)|dt≤ϵ2(∫01dt)1−(1/q) ×(∫01|1−λ−t|q|f′(ta+(1−t)(a+ϵ))|qdt)1/q +(b−a−ϵ)2(∫01dt)1−(1/q) ×(∫01|μ−t|q|f′(t(a+ϵ)+(1−t)b)|qdt)1/q=ϵ2(∫01|1−λ−t|q|f′(ta+(1−t)(a+ϵ))|qdt)1/q +(b−a−ϵ)2 ×(∫01|μ−t|q|f′(t(a+ϵ)+(1−t)b)|qdt)1/q.

By the convexity of |*f*′(*x*)|^*q*^ and Lemma 2, we have

(28) ∫01|1−λ−t|q|f′(ta+(1−t)(a+ϵ))|qdt=∫01|1−λ−t|q|f′((1−ϵ(1−t)b−a)a+ϵ(1−t)b−ab)|qdt≤∫01|1−λ−t|q  ×[(1−ϵ(1−t)b−a)|f′(a)|q+ϵ(1−t)b−a|f′(b)|q]dt=[(1−ϵb−a)|f′(a)|q+ϵb−a|f′(b)|q] ×∫01|1−λ−t|qdt   +[ϵb−a|f′(a)|q−ϵb−a|f′(b)|q] ×∫01t|1−λ−t|qdt   =[(1−ϵb−a)|f′(a)|q+ϵb−a|f′(b)|q] ×(1−λ)q+1+λq+1q+1 +[ϵb−a|f′(a)|q−ϵb−a|f′(b)|q] ×(1−λ)q+2+(q+2−λ)λq+1(q+1)(q+2)=[(1−λ)q+1+λq+1q+1−(ϵb−a)  ×(1+λ+q)(1−λ)q+1+λq+2(q+1)(q+2)]|f′(a)|q +[(ϵb−a)(1+λ+q)(1−λ)q+1+λq+2(q+1)(q+2)]|f′(b)|q,∫01|μ−t|q|f′(t(a+ϵ)+(1−t)b)|qdt=∫01|μ−t|q  ×|f′((t−ϵtb−a)a+(1−(t−ϵtb−a))b)|qdt≤∫01|μ−t|q  ×[(t−ϵtb−a)|f′(a)|q    +(1−(t−ϵtb−a))|f′(b)|q]dt=[(1−ϵb−a)|f′(a)|q−(1−ϵb−a)|f′(b)|q] ×∫01t|μ−t|qdt     +|f′(b)|q∫01|μ−t|qdt=[(1−ϵb−a)|f′(a)|q−(1−ϵb−a)|f′(b)|q] ×μq+2+(q+1+μ)(1−μ)q+1(q+1)(q+2) +|f′(b)|q(μq+1+(1−μ)q+1q+1)=[(1−ϵb−a)μq+2+(q+1+μ)(1−μ)q+1(q+1)(q+2)]|f′(a)|q +[μq+1+(1−μ)q+1q+1−(1−ϵb−a)  ×μq+2+(q+1+μ)(1−μ)q+1(q+1)(q+2)] ×|f′(b)|q.

Thus,

(29) |ϵλf(a)+(b−a−ϵ)μf(b) +[ϵ(1−λ)+(b−a−ϵ)(1−μ)] ×f(a+ϵ)−∫abf(x)dx|≤ϵ2{[(1−λ)q+1+λq+1q+1−(ϵb−a)     ×(1+λ+q)(1−λ)q+1+λq+2(q+1)(q+2)]|f′(a)|q    +[(ϵb−a)(1+λ+q)(1−λ)q+1+λq+2(q+1)(q+2)]     ×|f′(b)|q}1/q+(b−a−ϵ)2 ×{[(1−ϵb−a)μq+2+(q+1+μ)(1−μ)q+1(q+1)(q+2)]   ×|f′(a)|q    +[μq+1+(1−μ)q+1q+1−(1−ϵb−a)     ×μq+2+(q+1+μ)(1−μ)q+1(q+1)(q+2)]    ×|f′(b)|q}1/q.

This proof is completed.

It is easy to notice that if we put *ϵ* = (*b* − *a*)/2 in Theorem 10 then we get the following.

**Corollary 11 (see [8])**

Let *λ*,  *μ* ∈ [0,1] and let *f* : *I*⊆ℝ → ℝ be differentiable on *I*° and {*a*, *b*}⊆*I* with *a* < *b*. Assume that *f*′ ∈ *L*[*a*, *b*]. If |*f*′(*x*)|^*q*^ is convex on [*a*, *b*] with *q* ≥ 1, then

(30) |λf(a)+μf(b)2+2−λ−μ2f(a+b2)−1b−a∫abf(x)dx|≤b−a4(12(q+1)(q+2))1/q ×[((q+3−λ)(1−λ)q+1+(2q+4−λ)λq+1)|f′(a)|q    +((q+1+λ)(1−λ)q+1+λq+2)|f′(b)|q]1/q +b−a4(12(q+1)(q+2))1/q ×[((q+1+μ)(1−μ)q+1+μq+2)|f′(a)|q   +((q+3−μ)(1−μ)q+1+(2q+4−μ)μq+1)   ×|f′(b)|q]1/q.

One can easily check that if we put *ϵ* = (*b* − *a*)/3 in Theorem 10 then we get the following.

**Corollary 12**

Let *λ*,  *μ* ∈ [0,1] and let *f* : *I*⊆ℝ → ℝ be differentiable on *I*° and {*a*, *b*}⊆*I* with *a* < *b*. Assume that *f*′ ∈ *L*[*a*, *b*]. If |*f*′(*x*)|^*q*^ is convex on [*a*, *b*] with *q* ≥ 1, then

(31) |λf(a)+2μf(b)3+(1−λ3−2μ3)f(2a+b3) −1b−a∫abf(x)dx|≤b−a9 ×{(5+2q−λ)(1−λ)q+1+(6+3q−λ)λq+13(q+1)(q+2)|f′(a)|q    +(1+λ+q)(1−λ)q+1+λq+23(q+1)(q+2)|f′(b)|q}1/q +4(b−a)9 ×{2μq+2+2(q+1+μ)(1−μ)q+13(q+1)(q+2)|f′(a)|q   +(6+3q−2μ)μq+1+(4+q−2μ)(1−μ)q+13(q+1)(q+2)    ×|f′(b)|q}1/q.

One can easily check that if we put *ϵ* = 2(*b* − *a*)/3 in Theorem 10 then we get the following.

**Corollary 13**

Let *λ*,  *μ* ∈ [0,1] and let *f* : *I*⊆ℝ → ℝ be differentiable on *I*° and {*a*, *b*}⊆*I* with *a* < *b*. Assume that *f*′ ∈ *L*  [*a*, *b*]. If |*f*′(*x*)|^*q*^ is convex on [*a*, *b*] with *q* ≥ 1, then

(32) |2λf(a)+μf(b)3+(1−2λ3−μ3)f(a+2b3) −1b−a∫abf(x)dx|≤4(b−a)9 ×{(4−q−2λ)(1−λ)q+1+(6+3q−2λ)λq+13(q+1)(q+2)   ×|f′(a)|q+ 2(1+λ+q)(1−λ)q+1+2λq+23(q+1)(q+2)   ×|f′(b)|q}1/q +b−a9 ×{μq+2+(q+1+μ)(1−μ)q+13(q+1)(q+2)|f′(a)|q   +(6+3q−μ)μq+1+(5+2q−μ)(1−μ)q+13(q+1)(q+2)   ×|f′(b)|q}1/q.

It is easy to notice that if we put *λ* = *μ* = 0 in Theorem 10 then we get the following.

**Corollary 14**

Let *f* : *I*⊆ℝ → ℝ be differentiable on *I*° and {*a*, *b*}⊆*I* with *a* < *b*. Assume that *f*′ ∈ *L*[*a*, *b*] and 0 < *ϵ* < *b* − *a*. If |*f*′(*x*)|^*q*^ is convex on [*a*, *b*] with *q* ≥ 1, then

(33) |(b−a)f(a+ϵ)−∫abf(x)dx|≤ϵ2{(1q+1−ϵ(b−a)(q+2))|f′(a)|q    +ϵ(b−a)(q+2)|f′(b)|q}1/q +(b−a−ϵ)2 ×{(1−ϵb−a)(1q+2)|f′(a)|q    +(1q+1−(1−ϵb−a)(1q+2))|f′(b)|q}1/q.

It is easy to notice that if we put *λ* = *μ* = 1/2 in Theorem 10 then we get the following.

**Corollary 15**

Let *f* : *I*⊆ℝ → ℝ be differentiable on *I*° and {*a*, *b*}⊆*I* with *a* < *b*. Assume that *f*′ ∈ *L*[*a*, *b*] and 0 < *ϵ* < *b* − *a*. If |*f*′(*x*)|^*q*^ is convex on [*a*, *b*] with *q* ≥ 1, then

(34) |ϵf(a)+(b−a−ϵ)f(b)+(b−a)f(a+ϵ)2−∫abf(x)dx|≤ϵ22(1q+1)1/q{(1−ϵ2(b−a))|f′(a)|q          +ϵ2(b−a)|f′(b)|q}1/q +(b−a−ϵ)22(1q+1)1/q ×{(12−ϵ2(b−a))|f′(a)|q   +(12+ϵ2(b−a))|f′(b)|q}1/q.

It is easy to notice that if we put *λ* = *μ* = 1 in Theorem 10 then we get the following.

**Corollary 16**

Let *f* : *I*⊆ℝ → ℝ be differentiable on *I*° and {*a*, *b*}⊆*I* with *a* < *b*. Assume that *f*′ ∈ *L*[*a*, *b*] and 0 < *ϵ* < *b* − *a*. If |*f*′(*x*)|^*q*^ is convex on [*a*, *b*] with *q* ≥ 1, then

(35) |ϵf(a)+(b−a−ϵ)f(b)−∫abf(x)dx|≤ϵ2(1q+1)1/q{(1−ϵ(b−a)(q+2))|f′(a)|q          +ϵ(b−a)(q+2)|f′(b)|q}1/q +(b−a−ϵ)2(1q+1)1/q ×{(1−ϵb−a)(1q+2)|f′(a)|q   +(1−(1−ϵb−a)(1q+2))|f′(b)|q}1/q.

## 4. Applications

In this section, we suppose that {*s*, *q*}⊆[1, *∞*) and {*a*, *b*, *w*_*a*_, *w*_*b*_}⊆(0, *∞*) with *a* < *b*. Let 0 < *ϵ* < *b* − *a*.

The weighted arithmetic mean of data {*a*, *b*} with weight {*w*_*a*_, *w*_*b*_} is defined by

(36) $$\begin{array}{c} A\left(a,b;w_{a},w_{b}\right)=\frac{w_{a}a+w_{b}b}{w_{a}+w_{b}}. \end{array}$$

The weighted geometric mean of data {*a*, *b*} with weight {*w*_*a*_, *w*_*b*_} is defined by

(37) $$\begin{array}{c} G\left(a,b;w_{a},w_{b}\right)=a^{w_{a}/(w_{a}+w_{b})\;}b^{w_{b}/(w_{a}+w_{b})}. \end{array}$$

The generalized logarithmic mean of data {*a*, *b*} is defined by

(38) $$\begin{array}{c} L\left(a,b\right)={\left(\frac{b^{s+1}-a^{s+1}}{\left(s+1\right)\left(b-a\right)}\right)}^{1/s}. \end{array}$$

The identric mean of data {*a*, *b*} is defined by

(39) $$\begin{array}{c} I\left(a,b\right)=\frac{1}{e}{\left(\frac{b^{b}}{a^{a}}\right)}^{1/(b-a)}. \end{array}$$

Applying Corollary 7 with *f*(*x*) = *x* ^*s*^ on (0, *∞*), we get the following:

(40) 21−(1/q)(b−a)s|As(a,b;b−a−ϵ,ϵ)−Ls(a,b)|≤ϵ2(a(s−1)q2+ϵ(b(s−1)q−a(s−1)q)3(b−a))1/q +(b−a−ϵ)2(2a(s−1)q+b(s−1)q6+ϵ(b(s−1)q−a(s−1)q)3(b−a))1/q.

Applying Corollary 9 with *f*(*x*) = *x* ^*s*^ on (0, *∞*), we get the following:

(41) 21−(1/q)(b−a)s|A(as,bs;ϵ,b−a−ϵ)−Ls(a,b)|≤ϵ2(a(s−1)q3+ϵ(b(s−1)q−a(s−1)q)6(b−a))1/q +(b−a−ϵ)2(a(s−1)q+2b(s−1)q6+ϵ(b(s−1)q−a(s−1)q)6(b−a))1/q.

Applying Corollary 7 with *f*(*x*) = ln*x* on (0, *∞*), we get the following:

(42) 21−(1/q)(b−a)|ln(A(a,b;b−a−ϵ,ϵ)I(a,b))|  ≤ϵ2(a−q2+ϵ(b−q−a−q)3(b−a))1/q   +(b−a−ϵ)2(2a−q+b−q6+ϵ(b−q−a−q)3(b−a))1/q.

Applying Corollary 9 with *f*(*x*) = ln*x* on (0, *∞*), we get the following:

(43) 21−(1/q)(b−a)|ln(G(a,b;ϵ,b−a−ϵ)I(a,b))|  ≤ϵ2(a−q3+ϵ(b−q−a−q)6(b−a))1/q   +(b−a−ϵ)2(a−q+2b−q6+ϵ(b−q−a−q)6(b−a))1/q.

Applying Corollary 14 with *f*(*x*) = *x* ^*s*^ on (0, *∞*), we get the following:

(44) b−as|As(a,b;b−a−ϵ,ϵ)−Ls(a,b)|≤ϵ2(a(s−1)qq+1+ϵ(b(s−1)q−a(s−1)q)(b−a)(q+2))1/q +(b−a−ϵ)2 ×(b(s−1)qq+1+a(s−1)q−b(s−1)qq+2+ϵ(b(s−1)q−a(s−1)q)(b−a)(q+2))1/q.

Applying Corollary 16 with *f*(*x*) = *x* ^*s*^ on (0, *∞*), we get the following:

(45) (q+1)1/q(b−a)s|A(as,bs;ϵ,b−a−ϵ)−Ls(a,b)|≤ϵ2(a(s−1)q+ϵ(b(s−1)q−a(s−1)q)(b−a)(q+2))1/q +(b−a−ϵ)2 ×(b(s−1)q+a(s−1)q−b(s−1)qq+2+ϵ(b(s−1)q−a(s−1)q)(b−a)(q+2))1/q.

Applying Corollary 14 with *f*(*x*) = ln*x* on (0, *∞*), we get the following:

(46) (b−a)|ln(A(a,b;b−a−ϵ,ϵ)I(a,b))|≤ϵ2(a−qq+1+ϵ(b−q−a−q)(b−a)(q+2))1/q +(b−a−ϵ)2(b−qq+1+a−q−b−qq+2+ϵ(b−q−a−q)(b−a)(q+2))1/q.

Applying Corollary 16 with *f*(*x*) = ln*x* on (0, *∞*), we get the following:

(47) (q+1)1/q(b−a)|ln(G(a,b;ϵ,b−a−ϵ)I(a,b))|≤ϵ2(a−q+ϵ(b−q−a−q)(b−a)(q+2))1/q +(b−a−ϵ)2(b−q+a−q−b−qq+2+ϵ(b−q−a−q)(b−a)(q+2))1/q.
